# Intra-operative blink reflex neurophysiological monitoring in posterior skull base neurosurgery

**DOI:** 10.3389/fsurg.2025.1485459

**Published:** 2025-06-25

**Authors:** Michael W. Y. Lee, Shu-wah Chau

**Affiliations:** Department of Neurosurgery, Pamela Youde Nethersole Eastern Hospital, Hong Kong, Hong Kong SAR, China

**Keywords:** posterior skull base surgery, intra-operative neuro-monitoring, blink reflex, corneal reflex, predictive value

## Abstract

**Objectives:**

To enhance surgical safety with frequent intra-operative neuro-monitoring (IONM) of the blink reflex (BR) in posterior skull base neurosurgery.

**Background:**

There are reports stating the potential of facial nerve function preservation using BR IONM but none stating that it helps to protect corneal sensation and vision.

**Methods:**

A prospective cohort of 42 consecutive patients with lesions in proximity to the brainstem was operated between January 2021 and April 2024. BR, which is considered the electronic equivalent of the corneal reflex, is elicited by electrical stimulation of the supraorbital nerves. Recording electrodes for R1 responses are placed over orbicularis oculi muscle at the lower eyelid. BR signal loss or an amplitude drop by half or more in R1 from the baseline is regarded as a significant change in BR response. Pre-operative tumour volume and maximal midline shift (MLS) of the brainstem are measured, using readily identifiable anatomical landmarks, namely cerebral aqueduct, median sulcus of the fourth ventricle, and mid-point of interpeduncular fossa. Patients' intra-operative findings and post-operative clinical outcomes are correlated and reported.

**Results:**

BR IONM has a very high accuracy rate (96.7%) in predicting post-operative corneal complications, but can only be successfully done in 30 out of 42 patients (71.4%). There were 17 female and 13 male patients, with age ranging from 41 to 81 (mean 62.0). Ten underwent microvascular decompression and 20 had excision of tumours of size ranging from 1.1 to 81.6 cm^3^ (mean 21.6 cm^3^). For the tumour cases, Mann-Whitney U test for unpaired data showed statistically significant difference in tumour volume (*p* = 0.0016; 37.8 vs. 13.0 cm^3^) and maximal MLS of the brainstem (*p* = 0.0271; 8.1 vs. 4.8 mm) regarding the significant change in BR response. This suggests that BR change is a good sentinel indicator of how much brainstem neural tissue is mechanically stretched during excision of the tumour.

**Conclusions:**

Frequent IONM of BR is feasible, safe, and useful in the preservation of corneal sensation, especially for patients with large posterior fossa tumours and a distorted brainstem. For patients with the significant change in BR response, prompt referral to the ophthalmologist is useful for the prevention of keratitis and corneal ulceration.

## Introduction

To enhance the surgical safety of posterior skull base neurosurgery, besides the basic somatosensory evoked potential (SSEP), motor evoked potential (MEP), brainstem auditory evoked potential, and lower cranial nerves electromyography (EMG) monitoring, it is possible to perform direct brainstem stimulation for corticospinal tract motor response (1) and SSEP response (2) via scalp recordings. There are reports stating the potential of facial nerve function preservation (3) on frequent intra-operative neuro-monitoring (IONM) of the blink reflex (BR) but none stating that it helps to protect corneal sensation and vision.

### Corneal reflex, corneal sensation, and the neural pathway

The corneal reflex is an involuntary blinking of the eyelids, normally within 0.1 s, in response to the stimulation of the cornea. In conscious healthy subjects, stimulation should elicit both a direct and consensual response (blinking of the opposite eye). BR, as a relatively novel entity of IONM, is considered the electronic equivalent of this reflex.

Eric Kugelberg first described this reflex in humans in 1952 (4). The reflex and its pathway were described in detail by Jun Kimura in 2001 in conscious subjects (5). Vedran Deletis first reported how to record BR intra-operatively under general anaesthesia using propofol in 2009 with a successful rate of 86.2% (6). When the cornea is touched, an afferent impulse from either free nerve endings or mechanoreceptors within the corneal epithelium is sent by way of nasociliary nerve and its long ciliary nerve branches of the ophthalmic division of the trigeminal nerve (CN V) to the principal sensory nucleus (aka chief sensory nucleus) of CN V that is located in the rostral pons. Axons then project bilaterally to facial motor nucleus, which initiating the efferent motor response via the temporal and zygomatic branches of the facial nerve (CN VII), leading to the contraction of orbicularis oculi muscles and coordinated eye closure (blink). The rate of blinking is modulated by the blinking centre at the globus pallidus of the basal ganglia (7).

There are two stages to this reflex (8). Large-diameter myelinated A-beta fibers activate the initial movement of the eyelid on the ipsilateral side and provide the early response. Other smaller fiber types activate the late-stage reflex, which stimulates facial nerves bilaterally so that both eyes blink. Inputs from the secondary motor system (e.g., interpositus nucleus of the cerebellum, red nucleus, and reticular activating system) can also modulate this late-stage reflex (9).

Reduced corneal reflex and blinking can occur in various physiological and pathological conditions like old age, pregnancy (10), glaucoma, diabetes, herpetic keratitis, progressive supranuclear palsy, and Parkinson's disease (11, 12). Loss of sensory innervation of the cornea can result in a vision-threatening clinical condition known as neurotrophic keratopathy, which is characterized by reduced corneal sensation, tear film abnormalities and, in the most severe cases, persistent corneal epithelial defects, ulceration, and perforation of the stroma (12).

## Method

A prospective cohort of consecutive patients was operated for lesions in proximity to the brainstem under general anaesthesia between January 2021 and April 2024. Consent was obtained for IONM. Total intravenous anaesthesia (TIVA) was used. The body temperature and the haemodynamics of the patients as well as the infusion rate of anaesthetic agents, namely propofol at 10 mg/kg/h and remifentanil at 10 µg/kg/h, were kept stable throughout the period of IONM. The depth of anaesthesia was monitored with bispectral index (BIS), which was maintained at the range of 40–60. Muscle relaxants were not used, except for endotracheal intubation.

**Figure 1:**
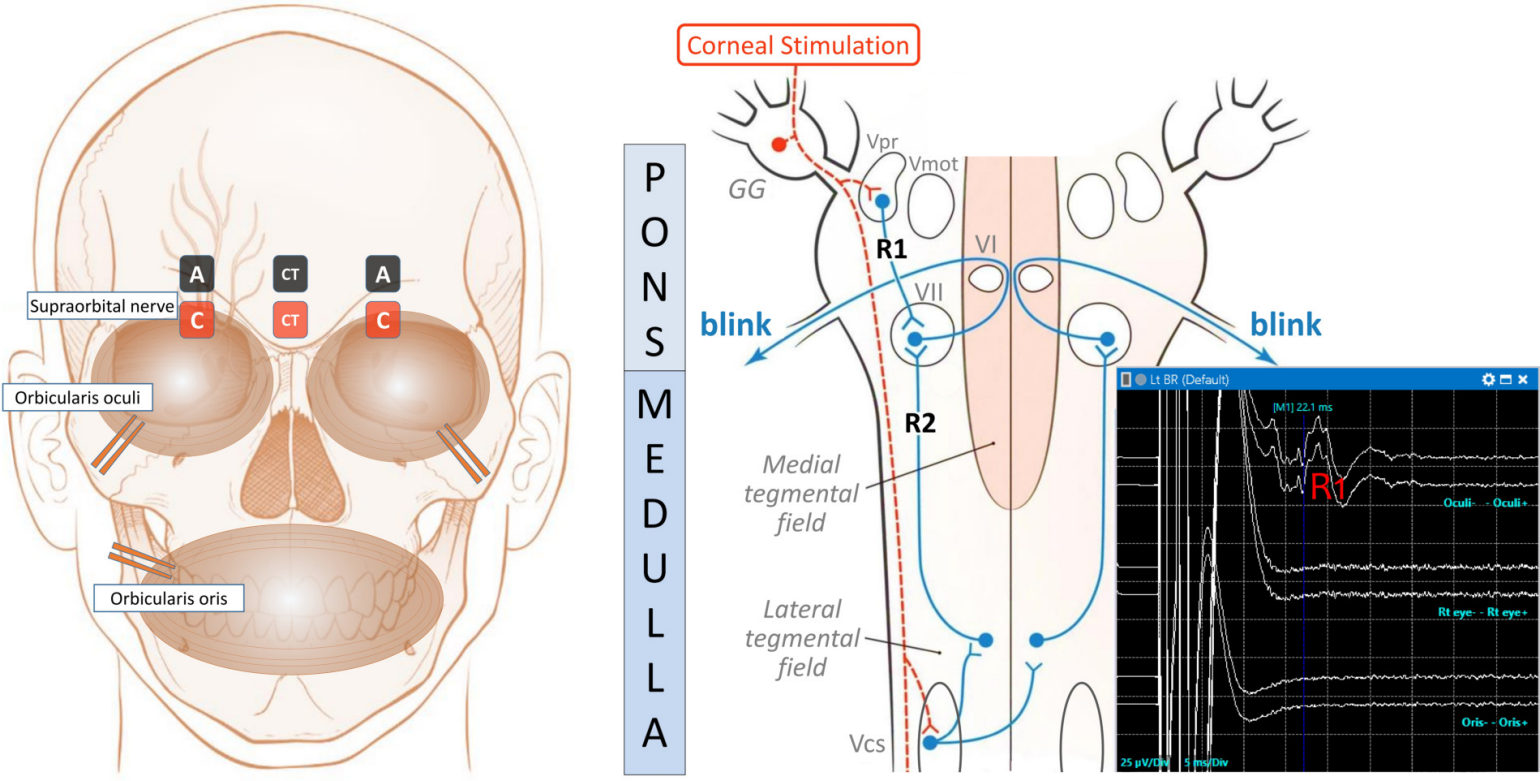
The setup, the pathway, and the typical BR IONM recordings. The left diagram shows the locations of the stimulating and recording electrodes for electrically induced blink reflexes in the ipsilateral orbicularis oculi muscle. A reference subdermal electrode is placed on the lateral aspect of the cheek. A pair of stimulating surface electrodes, spaced 2 cm apart, is used to stimulate the ipsilateral supraorbital nerve with the anode (A, black colour) positioned above the eyebrow, and the cathode (C, red colour) over the supraorbital notch. Recording needle electrodes are positioned at the ipsilateral orbicularis oculi muscle near the lower eyelid. In addition, a pair of recording control surface electrodes (CT) in the midline, along with needle electrodes at orbicularis oris muscles, act as the control to detect unintentional spread of stimulating electricity causing movement artefacts. The right schematic depicts the blink reflex pathways for R1 and R2 responses in conscious subjects. It is modified from Maciel et al. (14). The dotted orange line denotes the afferent pathway. The supraorbital nerve conveys the corneal stimulus to the Gasserian ganglion (GG) and then to the primary trigeminal nucleus (Vpr) and trigeminal motor nucleus (Vmot), where the ipsilateral R1 response is received via an oligosynaptic arc to the facial nucleus (VII). The supraorbital nerve also transports the afferent impulse to the caudal spinal trigeminal nucleus (Vcs) via the descending spinal tract of the trigeminal nerve in the lower brainstem. The efferent impulse (blue line) travels via the medullary pathway, which ascends bilaterally to connect to VII in the pons, looping around abducens nuclei (VI), and produces the R2 responses, which are clinically visible as bilateral blinking responses in conscious subjects. The right lower graph depicts the normal R1 response during left BR IONM under general anaesthesia, with a latency peak at 22.1 ms in the upper line. There should not be corresponding signals at that particular time in the middle and lower lines, which respectively, show the signals over the contralateral right eye via CT electrodes and orbicularis oris electrodes. They act as the control to detect movement artefacts. The scale is 25 µV/Div in the *Y*-axis for the amplitude, and 5 ms/Div on the *X*-axis for the time. R2 response is not elicitable under general anaesthesia.

BR is elicited by direct electrical stimulation of the supraorbital nerves, which produces a compound motor action potential response of the facial nerve, with two components, R1 and R2. The early R1 response is relatively synchronous and constant in duration and shape. The second response R2 is a late bilateral response, which corresponds to the clinically observable blink. The latter response is asynchronous, and it rapidly habituates and disappears bilaterally. Compressive lesions of trigeminal nerve, such as tumours or aneurysms, involve the afferent limb of the reflex arc and prolong the latency of ipsilateral R1 and bilateral R2. Facial nerve lesions affect the efferent limb of the blink reflex arc and delay the latency of ipsilateral R1 and R2. As previously reported, R2 response is not elicitable under general anaesthesia (13). The setup and the pathway for BR are depicted in Figure 1.

### Stimulation and recording parameters and diagnostic criteria

A stable R1 response is elicited using a train-of-four constant-current stimuli with an inter-stimulus interval of 2 ms, 300 μs pulse width and intensity of up to 40 mA at our hospital. Recording parameters are a time sweep of 50 ms and an amplification of 25–50 μV with a bandpass of 10–3 k Hz. The intensity required for optimal R1 response is found to be quite variable among individual patients due to inter-personal variability of response to anaesthesia as well as brain pathologies. In principle, the intensity of stimulation can be increased until there is a movement artefact in the EMG of contralateral orbicularis oculi or ipsilateral orbicularis oris. By convention, it is common to define an amplitude drop by half or more of a wave from the baseline as a significant change in IONM and thus we arbitrarily adopt it as our definition in the beginning. R1 signal loss or amplitude drop by half or more from the baseline is regarded as a significant change in BR response.

### Frequency of stimulation

**Figure 2:**
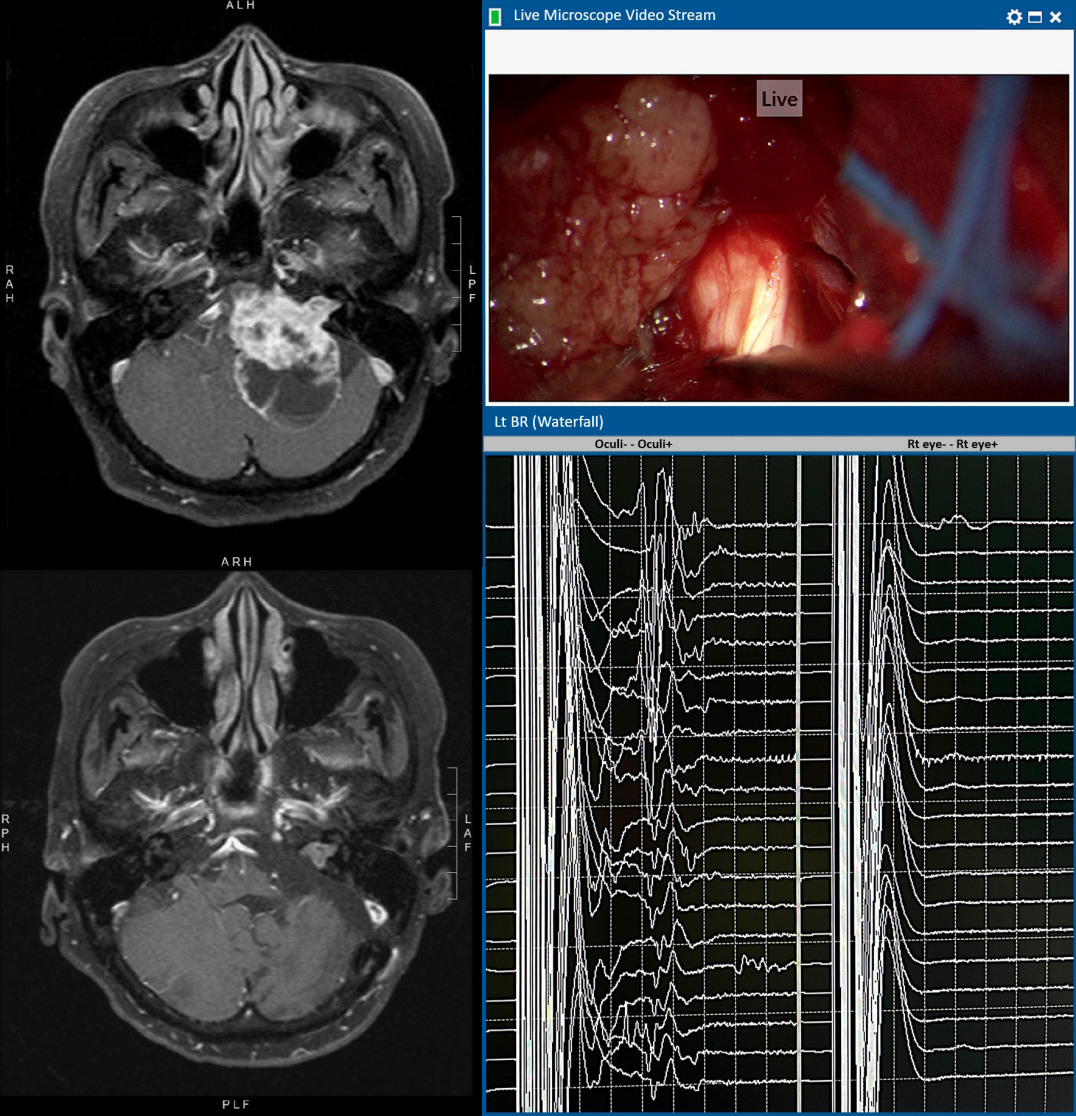
An illustrative case of left vestibular schwannoma with T1WI contrast MRI before (upper) and after (lower) operation. Intra-operative microscopic view shows that the cisternal portion of the trigeminal nerve is encased by the tumour. The left BR waterfall diagram shows preserved R1 waves throughout the operation. The tracing on the right shows no untoward movement artefacts detected by the control electrodes over the contralateral right eye. The facial nerve remained intact anatomically and physiologically (responsive to 0.1 mA stimulation) at the end of operation. Post-operatively, there is no facial nerve palsy and the corneal reflex is normal.

The BR can be monitored very frequently, as shown in the waterfall diagram in Figure 2. Stable BR tracings as the baseline are acquired before the opening of the skull bone. BR is recorded again as another reference after the opening of dura when there will be loss of cerebrospinal fluid and exposure of the brain to the cooler atmospheric air. It is well known that most IONM waveforms are temperature sensitive. At this point, the temperature of the operating field may gradually fall from normal core body temperature to room temperature unless warm normal saline irrigation is administered frequently. Then, during the micro-dissection, nonstop BR monitoring is performed, and it can be acquired as frequent as every minute. Finally, upon the closure of dura, the last BR will be recorded.

### Clinical assessment and metrics

In this study, standardized clinical assessment of corneal reflex and sensation is performed. The patient is approached from the side, out of the line of vision to avoid eliciting a menace response. The cornea is lightly touched with a thin strand of clean cotton. Blinking and tearing in that eye (direct corneal reflex) are observed as well as blinking over the other eye (consensual corneal reflex). The patient is asked to compare the sensation of touch over the cornea bilaterally. It is classified as a decreased response if there is a delay in blink or decreased sensation of touch over the cornea.

**Figure 3:**
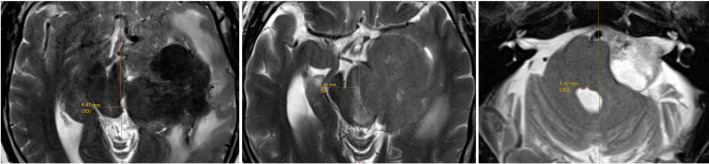
The maximal midline shift of brainstem is measured, using readily identifiable anatomical landmarks, namely cerebral aqueduct (left), mid-point of interpeduncular fossa, and median sulcus of the fourth ventricle in the T2WI MRI.

In addition, the facial nerve function is assessed according to the House-Brackmann grading system, pre-operatively, as well as on day one and at third month post-operatively. During the follow-up, the patients are assessed by clinicians who have access to the imaging and operation records. However, they do not know about the exact intra-operative BR findings that are archived by the IONM nurses during the operation. Pre-operative tumour volume is determined by volumetric measurement in a neuronavigation planning software with the tumour contouring done by the neurosurgeon. The maximal midline shift (MLS) of the brainstem is measured, using readily identifiable anatomical landmarks, namely cerebral aqueduct, mid-point of interpeduncular fossa, and median sulcus of the fourth ventricle in the T2-weighted images (T2WI) of the MRI (Figure 3). Patients' intra-operative findings and post-operative clinical outcomes are correlated and reported. Data are analysed using IBM SPSS Statistics version 29.0 using non-parametric tests with statistical significance set at *p* < 0.05.

## Results

There are 42 consecutive patients included in this study, and BR IONM is possible in 30 out of 42 patients (71.4%). There are 17 female and 13 male patients, with age ranging from 41 to 81 (mean 62.0). The average latency of R1 in 30 patients under general anaesthesia is 13.3 ms (range from 11.9–14.4 ms). The mean and median duration of follow-up are 95.3 and 89.9 weeks, respectively. Ten patients underwent microvascular decompression (trigeminal neuralgia in four and hemi-facial spasm in six) and 20 excision of tumours of size ranging from 1.1 to 81.6 cm^3^ (mean 21.6 cm^3^). For the tumour cases, Mann-Whitney U test for unpaired data show statistically significant difference in tumour volume (*p* = 0.0016; 37.8 vs. 13.0 cm^3^) and maximal MLS of the brainstem (*p* = 0.0271; 8.1 vs. 4.8 mm) regarding significant change in BR response. This suggests that BR change is a good sentinel indicator of how much brainstem neural tissue is mechanically stretched during the excision of the tumour.

### Diagnostic accuracy of BR

In the 30 patients with successful BR IONM, their BR is decreased significantly (four patients; 13.3%) or lost (two patients; 6.7%) in a total of six patients (20%) at the end of operation. For all patients with transient drop in R1 during the surgery, there are no new deficits if the R1 recovered at the end of operation. There is one (patient 11) with diminished but not reaching 50% amplitude drop in R1 and the patient does not have post-operative corneal sensation problem or ulceration. For the four patients (13.3%) with significant decrease in BR response, all have decreased ipsilateral corneal sensation and one has transient ulceration and keratopathy post-operatively, while for the two patients (6.7%) with BR lost, all have corneal ulcerations and exposure keratitis documented and managed by ophthalmologists. Considering all significant changes in BR and all post-operative corneal complications as post-operative neurological deficits, the statistic parameters of using BR response in detection of deficits are listed in Table 1 as follows.

**Table 1:** Diagnostic accuracy of BR in the detection of post-operative neurological deficits.

Statistic parameters	Value (%)	95% CI (%)
Sensitivity	100.0	54.0–100.0
Specificity	95.8	78.9–99.9
Positive predictive value	85.7	46.8–97.6
Negative predictive value	100.0	85.2–100.0
Accuracy	96.7	82.8–99.9

## Discussion

In a study of 30 healthy conscious adult subjects, the average latency of R1 is at 10.7 ms and R2 at 33.3 ms (15). Our average latency of R1 in 30 patients under general anaesthesia is 13.3 ms (range from 11.9 to 14.4 ms). The latency can vary with the level and method of anaesthesia.

### Merits and disadvantages of the various stimulation paradigms

For the stimulation parameters and recording techniques, there are slight variations in the literature, which are summarized in Table 2. Although TIVA is routinely used in IONM, there can be occasions that it is necessary to change the rate of infusion of anaesthetic agents. We use a train-of-four stimuli to overcome the inhibitory effect of the inadvertent fluctuation of anaesthetic drugs to ensure having a satisfactory R1 waveform in BR monitoring. The use of the train in stimulation is also reported by the Turkey (16) and Beijing (3) groups. We do not use halogenated agents, as in the Beijing (3) group, lest they can affect the other modalities of IONM.

**Table 2:** Merits and disadvantages of the various stimulation paradigms.

Study	Stimulation parameters	Stimulation and recording technique	Anaesthetic technique	Criteria	Successful rate and statistics
Turkey study ( (16)	A double train of 20–40 V intensity with an inter-train interval of 40–60 ms, an inter-stimulus interval of 2.5 ms, and a stimulus duration of 0.5 ms	Use of subdermal needles over supraorbital notch for stimulation and in the ipsilateral orbicularis oculi muscle for recording	TIVA (propofol 10 mg/kg/h and remifentanil 10 µg/kg/h)	R1 wave amplitude drop by more than 50% or total loss of BR considered as “permanent loss”	32 of 40 patients (80%) (sensitivity 75%, specificity 100%, and accuracy 96.8%)
Beijing study (3)	A single stimulation with four biphasic constant-current pulses. The pulse width is 200 μs, the pulse interval 2 ms, and the intensity 20–40 mA	Use of subdermal needles over supraorbital notch for stimulation and in the orbicularis oculi muscle for recording. The recording is performed after averaging two adjacent and stimulus-polarity-reversed responses	Propofol and halogenated agents, including low doses of sevoflurane (0.3 MAC)	Optimal cut-off value of BR amplitude deterioration (50.0%)	103 of 110 patients (93.6%) (sensitivity, 94.4%; specificity, 94.4%, and thus. accuracy 94.4%)
PYNEH study (Hong Kong)	Train-of-four constant-current stimuli with an inter-stimulus interval of 2 ms, 300 μs pulse width, and intensity of up to 40 mA	Use of surface electrodes over supraorbital nerves. Use of control electrodes. Recording by needle electrodes over ipsilateral orbicularis oculi	TIVA and BIS monitoring that are used in routine IONM	R1 wave amplitude decrease by 50% or more, or total loss of R1 wave	30 of 42 patients (71.4%) (sensitivity 100%, specificity 95.8%, and accuracy 96.7%)

We use surface electrodes over supraorbital nerves for more reliable stimulation, and this is similar to using surface electrodes for median nerve stimulation in SSEP. We find that subdermal needle electrodes can sometimes be far away from the nerve or inadvertently puncture and damage the nerve. The presence of control electrodes can detect and avoid inadvertent current spread that leads to false-positive signals that could be mistakenly recorded as genuine R1. This borrows the concept of detecting after-discharges in direct cortical stimulation in awake craniotomy that helps to determine the maximal safe and effective stimulation.

### Criteria for the significant change in BR IONM

We use the criteria of R1 wave amplitude decrease by 50% or more, or total loss of R1 wave as the criteria for significant change in BR IONM. This supports the criteria that were used by the Turkey (16) and Beijing (3) groups and has comparable satisfactory overall accuracies.

In one patient (No.26), there is no MLS despite the volume of the tumour being 3.2 cm^3^. This can sometimes happen when the tumour is mainly over the cistern and/or cerebellar region and not causing any shift in the brainstem. For example, for Koos grade I to III vestibular schwannoma, there will not be a significant shift in the brainstem in the pre-operative MRI. If, during excision of the tumour, using appropriate dynamic retraction after CSF is duly released from the cistern, it is possible to remove the tumour without causing a significant shift in the brainstem. Therefore, it is important to consider the parameter of maximal MLS in addition to the tumour volume when using BR IONM.

### Correlation of BR with facial MEP and facial palsy

Studies postulated that BR can predict post-operative facial palsy even better than facial corticobulbar MEP (FCoMEP) (13, 14). In our two patients (patients 8 and 15) with complete BR loss but intact FCoMEP, although they have corneal sensation disturbances and ulcerations, they do not have post-operative facial palsy. Both patients are managed early by ophthalmologists within post-operative two days and have a satisfactory outcome. We believe that BR may have a role that is more than another supplementary monitoring of facial nerve function as its neural pathway included both trigeminal and facial nerves pathways and R1 in BR is mainly related to the trigeminal pathway.

In addition, FCoMEP may cause extraneous movement interfering the microsurgery (3). This extraneous movement can also create false-positive recordings in intra-operative FCoMEP and BR monitoring, due to the vicinity of the facial muscles. In our study, we utilize the As Low As Reasonably Practicable principle in all our intra-operative stimulation, and in our experience, the pick-up rate of extraneous orbicularis oculi responses by FCoMEP is two out of 30 patients (6.7%). These are cases (case 11 and case 23 in Table 3) that increasing stimulation by no more than 20% from baseline was given for recording when the FCoMEPs show a reduction in amplitude. Furthermore, in our study, we stimulate the ipsilateral supraorbital nerve while using a pair of control electrodes placed in the midline to detect and avoid unwanted movement artefacts. As a result, there is no interference with microsurgery at all when utilizing BR.

**Table 3:** Clinical and radiological characteristics of the patient cohort.

No.	Gender	Age	Diagnosis	Side	Volume (cm^3^)	Maximal MLS (mm)	Pre-operative neurological exam		Intra-operative findings		Post-operative outcome at 3 months		
							Corneal reflex	VII (HBS)	BR	FCoMEP	Corneal reflex	VII (HBS)	Ophthalmologist findings
1	F	67	TN	R	na	0.0	N	1	N	N	N	1	N
2	F	53	TN	R	na	0.0	N	1	N	N	N	1	N
3	F	53	HFS	L	na	0.0	N	1	N	N	N	1	N
4	M	64	VS	L	7.37	5.5	N	1	N	N	N	1	N
5	F	60	HFS	R	na	0.0	N	1	N	N	N	1	N
6	M	81	VS	L	28.56	8.3	N	1	Significant ↓↓	N	↓↓	3	Corneal hypoesthesia
7	F	57	HFS	R	na	0.0	N	1	N	N	N	1	N
8	M	64	Petrous meningioma (WHO grade 2)	L	81.62	6.7	↓	1	lost	N	lost	1	Exposure keratopathy
9	M	68	4th ventricular PFB ependymoma	Bilateral	2.99	3.1	N	1	N	N	N	1	N
10	F	65	Medial sphenoidal ridge meningioma (WHO grade 2)	R	9.24	1.0	N	1	N	N	N	1	N
11	M	65	Petrous meningioma (WHO grade 2)	L	46.64	8.6	N	1	Insignificant ↓	Significant ↓↓	N	1	N
12	M	59	VS	R	24.83	6.8	N	3	Significant ↓↓	Same as baseline	↓	3, no change	Corneal hypoesthesia
13	F	69	Medial sphenoidal ridge meningioma (WHO grade 1)	L	56.45	6.0	N	1	N	N	N	1	N
14	F	63	Petrous meningioma (WHO grade 1)	R	1.11	1.0	N	1	N	N	N	1	N
15	M	65	VS	R	28.42	7.0	↓	1	lost	N	lost	1	Exposure keratopathy
16	F	62	HFS	R	na	0.0	N	1	N	N	N	1	N
17	F	57	Petroclival meningioma (WHO grade 1)	L	10.80	5.0	N	1	N	N	N	1	N
18	F	57	TN	R	na	0.0	N	1	N	N	N	1	N
19	F	41	VS	L	1.55	1.8	N	1	N	N	N	1	N
20	F	65	VS	R	9.66	5.8	N	1	N	N	N	1	N
21	F	67	HFS	R	na	0.0	N	1	N	N	N	1	N
22	M	71	Trigeminal schwannoma	R	29.95	9.1	N	1	N	N	N	1	N
23	M	58	VS	R	38.12	11.8	↓	1	Significant ↓↓	Significant ↓↓	↓	3	Transient corneal ulceration and keratopathy
24	M	48	HFS	R	na	0.0	N	1	N	N	N	1	N
25	M	52	VS	R	9.10	7.9	N	1	N	N	N	1	N
26	F	58	VS	L	3.20	0.0	N	1	N	N	N	1	N
27	F	68	TN	R	na	0.0	N	1	N	N	N	1	N
28	M	79	Petrous meningioma (WHO grade 2)	L	16.07	7.5	N	1	Significant ↓↓	N	↓	3	Corneal hypoesthesia
29	M	68	VS	L	10.92	8.4	N	1	N	N	N	1	N
30	F	56	VS	R	16.24	8.0	N	1	N	N	N	1	N

F, female; M, male; TN, trigeminal neuralgia; HFS, hemi-facial spasm; VS, vestibular schwannoma; R, right; L, left; N, normal; na, not applicable; ↓, decreased; MLS, midline shift; BR, blink reflex; FCoMEP, facial corticobulbar motor evoked potential; HBS, House-Brackmann scale.

With regard to the impact on management, for patients with significant change in BR, we suggest early involvement of ophthalmologists and meticulous preventive eye care measures such as temporary tarsorrhaphy to avoid undesirable complications like exposure keratitis, ulceration, blindness, and other visual disturbances.

### Limitations of the current study

The current study presents a prospective cohort of 42 consecutive patients with posterior skull base neurosurgery using frequent BR IONM for monitoring of the corneal reflex and sensation under general anaesthesia. Given the variability in patient outcomes and the complexity of skull surgeries, this relatively small sample size may limit the study's statistical power to detect significant associations or draw definitive conclusions. Nevertheless, we reported statistically significant findings as well as the diagnostic accuracy of BR using our described method of electrode placement, stimulation and recording parameters, and diagnostic criteria. We hope that our report of the novel use of this technique may arouse the interest of the skull base neurosurgery community and possibly lead to further verification of our findings in a larger cohort.

## Conclusion

Frequent BR IONM is feasible, safe, and useful in the preservation of corneal sensation, especially for patients with large posterior fossa tumours and a distorted brainstem. BR IONM has a very high accuracy rate (96.7%), but can only be successfully done in 71.4% of our patients. This can be related to absent or diminished BR due to various reasons. For patients with significant change in BR response, prompt referral to the ophthalmologist is useful for the prevention of keratitis and corneal ulceration.

## Data Availability

The raw data supporting the conclusions of this article will be made available by the authors without undue reservation.
